# Assessment and ascertainment in psychiatric molecular genetics: challenges and opportunities for cross-disorder research

**DOI:** 10.1038/s41380-024-02878-x

**Published:** 2024-12-27

**Authors:** Na Cai, Brad Verhulst, Ole A. Andreassen, Jan Buitelaar, Howard J. Edenberg, John M. Hettema, Michael Gandal, Andrew Grotzinger, Katherine Jonas, Phil Lee, Travis T. Mallard, Manuel Mattheisen, Michael C. Neale, John I. Nurnberger, Wouter J. Peyrot, Elliot M. Tucker-Drob, Jordan W. Smoller, Kenneth S. Kendler

**Affiliations:** 1 Helmholtz Pioneer Campus, Helmholtz Munich, Neuherberg, Germany; 2 Computational Health Centre, Helmholtz Munich, Neuherberg, Germany; 3 School of Medicine and Health, Technical University of Munich, Munich, Germany; 4 Department of Psychiatry and Behavioral Sciences, Texas A&M University, College Station, TX USA; 5 Centre of Precision Psychiatry, University of Oslo, Oslo, Norway; 6 Division of Mental Health and Addiction, Oslo University Hospital, Oslo, Norway; 7 KG Jebsen Centre for Neurodevelopmental disorders, University of Oslo, Oslo, Norway; 8 Department of Cognitive Neuroscience, Donders Institute for Brain, Cognition and Behavior, Radboud University Medical Center, Nijmegen, The Netherlands; 9 Karakter Child and Adolescent University Center, Nijmegen, The Netherlands; 10 Department of Biochemistry and Molecular Biology, Indiana University School of Medicine, Indianapolis, IN USA; 11 Department of Medical and Molecular Genetics, Indiana University School of Medicine, Indianapolis, IN USA; 12 Virginia Institute for Psychiatric and Behavioral Genetics, Virginia Commonwealth University, Richmond, VA USA; 13 Departments of Psychiatry and Genetics, University of Pennsylvania, Philadelphia, PA USA; 14 Lifespan Brain Institute at Penn Med and the Children’s Hospital of Philadelphia, Philadelphia, PA USA; 15 Institute for Behavioral Genetics, University of Colorado Boulder, Boulder, CO USA; 16 Department of Psychology and Neuroscience, University of Colorado Boulder, Boulder, CO USA; 17 Department of Psychiatry & Behavioral Health, Stony Brook University, Stony Brook, NY USA; 18 Center for Genomic Medicine, Massachusetts General Hospital, Boston, MA USA; 19 Department of Psychiatry, Harvard Medical School, Boston, MA USA; 20 Center for Precision Psychiatry, Department of Psychiatry, Massachusetts General Hospital, Boston, MA USA; 21 Psychiatric and Neurodevelopmental Genetics Unit, Center for Genomic Medicine, Massachusetts General Hospital, Boston, MA USA; 22 Department of Community Health and Epidemiology and Faculty of Computer Science, Dalhousie University, Halifax, NS Canada; 23 Institute of Psychiatric Phenomics and Genomics (IPPG), University Hospital of Munich, Munich, Germany; 24 Department of Biomedicine, Aarhus University, Aarhus, Denmark; 25 Department of Psychiatry, Virginia Commonwealth University, Richmond, VA USA; 26 Department of Psychiatry, Indiana University School of Medicine, Indianapolis, IN USA; 27 Stark Neurosciences Research Institute, Indiana University School of Medicine, Indianapolis, IN USA; 28 Department of Psychiatry, Amsterdam UMC, Vrije Universiteit, Amsterdam, The Netherlands; 29 Amsterdam Public Health, Amsterdam UMC, Vrije Universiteit, Amsterdam, The Netherlands; 30 Department of Psychology, University of Texas at Austin, Austin, TX USA; 31 Stanley Center for Psychiatric Research, Broad Institute of MIT and Harvard, Cambridge, MA USA

**Keywords:** Genetics, Diagnostic markers

## Abstract

Psychiatric disorders are highly comorbid, heritable, and genetically correlated [1–4]. The primary objective of cross-disorder psychiatric genetics research is to identify and characterize both the shared genetic factors that contribute to convergent disease etiologies and the unique genetic factors that distinguish between disorders [4, 5]. This information can illuminate the biological mechanisms underlying comorbid presentations of psychopathology, improve nosology and prediction of illness risk and trajectories, and aid the development of more effective and targeted interventions. In this review we discuss how estimates of comorbidity and identification of shared genetic loci between disorders can be influenced by how disorders are measured (phenotypic assessment) and the inclusion or exclusion criteria in individual genetic studies (sample ascertainment). Specifically, the depth of measurement, source of diagnosis, and time frame of disease trajectory have major implications for the clinical validity of the assessed phenotypes. Further, biases introduced in the ascertainment of both cases and controls can inflate or reduce estimates of genetic correlations. The impact of these design choices may have important implications for large meta-analyses of cohorts from diverse populations that use different forms of assessment and inclusion criteria, and subsequent cross-disorder analyses thereof. We review how assessment and ascertainment affect genetic findings in both univariate and multivariate analyses and conclude with recommendations for addressing them in future research.

## Introduction

The comorbidity between psychiatric disorders stems, at least in part, from overlapping genetic factors. Understanding the genetic etiology of psychiatric outcomes can illuminate the common biological mechanisms that contribute to comorbid presentations of psychopathology, delineate distinct psychiatric disorders, and aid the development of more effective and targeted interventions. We focus on binary diagnoses of psychiatric disorders to link the implications of our recommendations to clinically validated outcomes and remain consistent with existing psychiatric genetics research. It could be argued that dimensional characterizations of psychopathology have more statistical power and are more capable of dissecting symptom heterogeneity. Nevertheless, we emphasize the clinical validity of categorical diagnoses which have been used extensively in psychiatric genetic analyses to situate our discussion within the context of large genomic initiatives such as the Psychiatric Genomics Consortium (PGC). Our goal in this review is to summarize current research and perspectives on how assessment and ascertainment strategies impact genetic findings for both individual disorders (Table 1) and, in turn, cross-disorder genetic sharing and the genetics of comorbidity and disease trajectories (Table 2). After examining the impact of various common assessment and ascertainment methods, we conclude with recommendations for collecting new genomic data and conducting rigorous genetic analyses in the future.

**Table 1:** Summary of assessment and ascertainment strategies for individual psychiatric disorder diagnoses in case-control cohorts, volunteer-based biobanks, and electronic health records (EHRs) or registries.

Aspects	Domains	Case-control	Volunteer-based biobanks	Electronic health records (EHRs), registries
General	Sample size	100–1000 s	100,000 s	Millions
	Sampling	Hospital-based clinical samples may be more severe than community samples	Volunteers may be healthier and have higher socioeconomic status than population average	Individuals accessing health system may be more unwell and have more comorbidity than population average
Assessment	Depth	Usually deep, using fully structured interviews	Varies, ranging from shallow single-item self-reports of illness, diagnosis, or treatment to deeper self-administered symptom-based questionnaires	Usually a diagnostic code, doctors’ notes may be available, secondary information such as prescription codes may be available
	Source	Clinicians or trained interviewers assess patients, parents, and/or teachers	Self-reported questionnaires (online or paper), or interviews at a collection center	Clinician, nurses, insurance codes
	Timeframe	Cross-sectional	Cross-sectional, sometimes repeated assessments through voluntary recontact	Longitudinal, length of follow-up based on the frequency of contact with the health system
Ascertainment	Case ascertainment	Usually severe, may have screened and excluded other psychiatric conditions	Cases may be less severe with less loss of function; comorbidity information may be available	Cases may be more severe and comorbid, there usually is comorbidity information
	Control ascertainment	Usually screened, may exclude cases of other psychiatric conditions	Sometimes screened, but may contain some cases due to mis-reporting or memory biases	Usually unscreened, may contain some cases due to diagnostic biases

**Table 2:** Sources of biases in individual disorder genetics, their potential impact on cross disorder genetic studies, mitigating factors in analyses, and recommendations for future data collection taking these biases and their effects into account.

Source of bias		Potential effect on cross-disorder analysis	Strategies for mitigation	Future data collection recommendations
Depth	Diagnoses from shallow phenotyping assessments are usually less valid, incurring high levels of misdiagnosis that may not be random	Nonspecific genetic effects on individual disorders; inflated rG between disorders in the case of bidirectional misdiagnosis between two disorders; mixture of unknown biases in shared genetic effects identified between two disorders	Assess the replicability or heterogeneity of effects across assessment strategies; assess specificity of polygenic risk scores (PRS); methods to combine different measures while maintaining specificity: (1) LT-FH, (2) MTAG, (3) Genomic SEM, (4) imputation	Use deep phenotyping where possible; use brief self-report versions of full diagnostic criteria if only shallow phenotyping is possible; repeated assessments; expand collection of data to include non-DSM symptoms and non-diagnostic information to supplement clinical characteristics obtained
Source	Diagnoses made by different sources may have different levels of validity and biases; concordance between diagnoses made by different sources may differ by disorder			Assessments by trained mental health professionals who are familiar with the relevant symptomatology of individual disorders and their usual comorbidities; establish quality control of interviewers; complement interviews with doctors’ notes, prescription and other medical records. For online assessment, avoid single item screens and use brief measures that assess full diagnostic criteria
Timeframe	Diagnoses made in different timeframes may reflect subsyndromal states or lifetime liabilities to disorder; effects compound with those from source of info and depth of assessment; effect of timeframe may be disorder specific			Focus on diagnoses made with assessments of lifetime, not current, symptoms; repeated assessments
EHRs	Only capture those who interact with the healthcare system, who may be unhealthier while having higher socioeconomic status than the population	Genetics of individual disorders unrepresentative of disorder in the population; rG between disorders may be inflated if they share common ascertainment patterns; disorders with different levels of dysfunction may not share ascertainment patterns, leading to deflation in their rGs	Epidemiologically verify disorder validity using known relationships with non-clinical factors; inverse probability (IP) weightings to improve representativeness (up-weighting participants with features identified to be associated with lower participation)	Collection of non-clinical epidemiological information, collection of repeated measurements; for international studies, pay attention to translations of assessment instruments and, when possible, assess your success though measurement non-invariance techniques
Biobanks	Only capture those who volunteer to participate, who may be healthier, better educated, and of higher socioeconomic status than the population			
Case-control cohorts	Biased toward treatment-seeking, high severity, excess comorbidity, and treatment non-responsiveness	Exaggerated case-control differences; may deflate rG proportional between disorders depending on genetic sharing between high severity forms of both disorders	Assess extent of biases to understand differences between inclusion and exclusion criteria	Design an ascertainment frame for cases that avoids oversampling of those with severe and/or treatment resistant illness
	The exclusion of prior or lifetime diagnoses of other disorders	Deflate rG between disorders as explicitly removed those with shared genetics		Collect information on prior or lifetime diagnosis of other disorders to assess their impact on individual disorder liability and cross disorder sharing
Screened “super-normal” controls	Screened for the disorder being studied and other psychiatric disorders not screened out in cases	Exaggerates case-control differences; produces spurious co-aggregation between disorders; inflates rG proportional to the population prevalence of the two disorders	Predicting disorder liability for unscreened controls	Use representative (not super-normal) controls
Unscreened controls	Containing cases of the target disorder at approximately the population prevalence	Genetic associations of the GWAS are downwardly biased, with the magnitude of the bias increasing for more prevalent disorders in the population, affecting rG between disorders accordingly		Screen controls as much as possible

## Assessment of individual psychiatric disorders

Diagnoses of psychiatric disorders used in genomic studies are obtained from a variety of research designs: clinician (or trained-research staff) administered structured interviews, self-administered questionnaires on current, and lifetime worst-episode symptoms [6, 7], self-reports of a prior or current diagnosis or treatment [7], and diagnostic codes from electronic health records (EHRs) [8–10] or registries [11]. The reliability and validity of psychiatric diagnoses are a function of variation in these assessment strategies within three primary domains.

The first domain is the depth of clinical detail with which a diagnosis is based. Structured interviews epitomize “deep” phenotyping, providing rich information on the clinical characteristics used to assign a diagnosis. Established instruments, such as the Structured Clinical Interview for DSM (SCID) [12] or the Composite International Diagnostic Interview (CIDI) [13], assess all symptoms, functional impairment, and exclusion criteria required for a DSM [14] or ICD [15] diagnosis. Some studies use the Operational Criteria Checklist [16], which leverages multiple operational diagnostic systems to enable consensus best-estimate procedures [17]. Such “deep” phenotyping was widely applied in the initial phases of the Psychiatric Genomic Consortium (PGC) meta-analyses [18–20]. This approach results in diagnoses that reflect current clinical standards and enables investigations into clinical heterogeneity. Supplementing “deep” phenotyping with assessments of other relevant psychiatric disorders, personality traits [21], early life factors [22], and stressful life events [23] further enables investigations into psychological and environmental correlates of disorders [24]. Conversely, “shallow” assessments allow us to quickly obtain large, inexpensive samples, accelerating gene-discovery efforts by increasing statistical power. However, shallow assessments, such as very short screening tools (one to four item scales) [25], while correlated with structured interviews, often yield high false positive rates [25] jeopardizing their clinical validity. Between these extremes exists a spectrum of assessment methods that vary in their depth, including self-reported symptom-based questionnaires, self-reported professional diagnoses or treatment, diagnostic codes (ICD9, ICD10), hospital visits, prescription records, and insurance claims based on clinical assessments from EHRs. These assessment techniques vary wildly in their reliability and validity. For example, diagnoses based on brief internet surveys may have questionable clinical validity while, for some disorders, online assessment instruments that assess a full set of diagnostic criteria have better psychometric properties [26]. Alternatively, those derived from prescriptions of restricted drugs, such as clozapine for treatment-resistant schizophrenia (SZ), can offer highly valid diagnoses [18, 27, 28]. Lower levels of reliability and clinical validity of shallow assessments may result in the misclassification of sub-clinical respondents as cases, influencing both genetic associations with the primary diagnosis and subsequent genetic correlations with comorbid conditions [29–32].

The second domain is the source of the assessment. Assessments of psychiatric disorders may come from clinicians (e.g., psychiatrists, other physicians, psychologists), trained research staff, self-reports, and relative or teacher reports [33–37]. The reliability and clinical validity of the psychiatric assessments vary as a function of the expertise of the interviewer, especially if the training or background of the interviewers enable them to create a sense of safety or rapport that allows the respondent to answer honestly, even for embarrassing topics. Consistency between trained psychiatrists and primary care physicians varies but is often high with repeated examinations [38, 39]. Diagnostic interviews conducted by trained research staff using semi-structured interviews such as the CIDI have been shown to have high validity when compared with structured interviews by clinicians [40]. However, diagnoses based on clinician ratings show significant differences from those relying on self-report [41, 42], with self-reports often being more severe [43, 44]. Furthermore, genetic analyses find that self-reports [45, 46] capture non-specific genetic effects and miss a significant portion of the genetic contributions to clinically defined disorders [45–48]. It remains unknown whether differences in validity between clinical and self-report diagnoses can be compensated for by repeated assessments [49]. Notably, the validity of self-reports can be influenced by disease-, symptom- and individual-specific factors that depend on a respondent’s comprehension of the questionnaire, motivation, and ability to answer accurately [50]. These self-report biases may be related to personality traits [51] or specific psychiatric symptoms [52] (which may influence disorder vulnerability), potentially impacting the reliability and generalizability of research findings.

The third domain is the time frame of the assessment. Genomic studies have started to explore how genetic variants affect temporal features of psychiatric disorders. Notably, lifetime diagnoses tend to be more heritable than current diagnoses [53, 54]. Genetic analyses demonstrate that self-reported current symptoms assessed by the Patient Health Questionnaire 9 are more reflective of subsyndromal dysphoria that is related to stressful life events and neuroticism, while self-reported worst-episode symptoms assessed through the CIDI Short Form [55] show greater genetic sharing with major depressive disorder (MDD). This suggests using current symptoms for identifying genetic contributions to disorders is likely to result in findings with low specificity that may be best limited to use in making current diagnoses. Alternatively, lifetime symptoms and diagnoses, may be modestly affected by inaccurate recollections, or other features of state-dependent memory [56]. The combination of over- and under-reporting due to selective recall introduces an unpredictable mixture of biases that depend on the lifetime prevalence of subsyndromal symptoms and is confounded with the source of the information (i.e., self-report vs clinician assessment) [57]. Genomic studies have started to explore how genetic variants affect other temporal features of psychiatric disorders. For example, age at onset or recurrence can reflect differences in genetic risk [58–60], and the timing of assessment relative to disorder onset can substantially affect genetic findings. More targeted analyses that isolate the effects of different time scale factors are needed.

As effect sizes of associations between individual genetic variants and psychiatric phenotypes are usually small, we need large sample sizes to obtain reproducible results. This means meta-analyzing data spanning all three assessment domains. The justification for integrating potentially heterogeneous phenotypes is usually based on high genetic correlations (rGs) between them. However, there are notable differences in the rGs among assessments of different disorders. The reported rGs between SZ samples collected through different means and populations are high (>0.9) [61, 62] while the rGs between MDD samples are as low as 0.59 [10]. Ignoring this variability may skew our understanding of the genetic architecture of individual disorders, rGs between disorders, and downstream analyses such as tissue-enrichment of the SNP-based heritability (h^2^_SNP_), and prioritization of GWAS findings for fine-mapping and drug-target identification.

How strictly should individual or cross-disorder psychiatric genetics research rely on deep, clinician-assessed diagnoses based on established DSM criteria rather than shallow, self-reported symptoms or EHRs? The DSM is neither perfect nor immutable and is periodically revised based on advances in the understanding of the etiology of the disorders. DSM criteria do not, nor are they designed to, exhaustively capture the diagnostic complexity of any specific disorder [63, 64]. However, DSM-based diagnoses correspond with current best-practice patient care, providing reliable assessments and underscoring their clinical validity for translating research into beneficial patient outcomes. Nevertheless, dichotomizing individuals into cases and controls discards potentially valuable information regarding disease severity thereby potentially reducing the power to detect genetic associations. Alternatively, self-reported questionnaires are less expensive to administer, allowing researchers to collect substantially more data, increasing statistical power at the potential cost of clinical reliability and validity. Thus, it is important to consider supplementing data on current diagnostic criteria with additional measures, such as self-reports, to identify additional factors that may play an important role for refining the diagnostic formulations and subtypes of psychiatric disorders. In many ways deep, clinician-assessed diagnoses compliment shallow, self-reported measures, and vice versa. The challenge will be to integrate seemingly disparate assessment methods in a way that maximizes the clinical validity of structured interviews and the recruitment potential of self-reported measures. As such, understanding how different assessment procedures affect empirical findings will streamline the integration of genomic evidence into future DSM revisions [65], with the goal of using epistemic iteration to refine diagnostic criteria [66, 67].

## Ascertaining cases and controls for individual disorders

### Case ascertainment

Strategies for identifying and recruiting individuals who meet diagnostic criteria for a psychiatric disorder can influence genetic associations and their interpretations [68]. Ascertainment for genomic studies primarily occurs in three forms: targeted recruitments of cases with a specific disorder from clinical or research settings, sampling from EHRs, and population-based sampling. While ascertainment strategies are theoretically independent of assessment methods and the prevalence of the target phenotype, practical constraints can confound these design factors.

Early in the psychiatric GWAS era, genomic studies primarily relied on targeted recruitments, requiring the coordination of networks of mental health professionals to screen patients for a target disorder, typically employing deep phenotyping [69–71]. This strategy was effective for the initial GWAS of rare disorders, particularly SZ [72] and bipolar disorder [73] (BD). Importantly, participants recruited from clinical settings frequently exhibit more severe illness than their counterparts in EHR and population-based studies [74–76]. Targeted approaches are typically the best way to obtain large numbers of cases of relatively rare disorders [77, 78]. One concern with this approach is whether such samples are representative, or biased toward treatment-seeking, severity, excess comorbidity, and/or treatment non-responsiveness. In addition, the exclusion of cases with other comorbid disorders (common among core PGC cohorts) likely affects its profile of genetic sharing, dependent on the patterns of comorbidity. Nonetheless, these ascertainment techniques, underscored by rigorous assessment methods, contributed to the success of the early PGC GWAS efforts.

National registries [79, 80] and EHRs [81–83] record healthcare information for everyone in their catchment, making them effective ascertainment strategies for identifying common and rare disorders. Patient diagnostic codes available through these resources can, in some instances, have high validity. For example, several follow-up clinical studies of cases [84, 85] of SZ [86, 87], BD [31], and obsessive compulsive disorder (OCD) [88] in Swedish and Danish registries and American EHRs have demonstrated strong validation against DSM criteria. Some EHRs have comprehensive doctors’ notes from individual interviews, which – if carefully coded – can augment case-control outcomes for genetic analyses [32, 89, 90]. Diagnostic data from EHRs and registries, however, can be heterogeneous. First, some healthcare systems use billing codes and base insurance claims or reimbursements on diagnostic assignment, while others do not. These incentive structures can create systematic biases in code assignment [91, 92]. Second, diagnoses inferred from administrative sources (e.g., pharmaceutical records) are indirect, adding uncertainty into the “case” phenotype. Third, different diagnostic biases, such as those related to search satisfaction (leading to underdiagnosis of comorbidities) and diagnostic momentum (sticking to a previous or working diagnosis even when it is erroneous) may differentially affect specific psychiatric disorders [44, 93].

EHRs and registries, however, may not be representative, capturing only those who interact with the healthcare system, and may oversample individuals with comorbidities and increased access to healthcare [94, 95]. This results in a disproportionate number of unhealthy individuals in EHRs, depending on the specific psychiatric disorder [96]. Further, EHRs based on insurance records, common in the US, may bias the presence of diagnosis or diagnostic classifications due to variable mobility, socioeconomic status and access to healthcare. This ascertainment problem can lead to biased estimates of polygenic score effect sizes. They EHRs also substantially under-represent early-onset disorders such as autism spectrum disorder [97], especially in females, though correlates later-in-life may be informative [84, 85]. These ascertainment problems affect the representativeness of the samples and can significantly affect cross-disorder genetic results by potentially biasing genetic analyses [96]. Finally, registries or EHRs may not contain information that provides a psychosocial context for the patient’s illness. Nonetheless, innovative ways to utilize EHR and registry data have potential for case identification [98–100].

Population-based biobanks are a common non-targeted means to collect data on psychiatric disorders [101] which have proven particularly useful for genomic analyses of common psychiatric disorders that are amenable to large-scale data collection using self-administered questionnaires with varying depth and time frames of assessment [45, 55]. However, population-based recruitment is sensitive to healthy volunteer biases. For example, the UK Biobank [102] invited approximately 9 million individuals to participate but only recruited 500,000 respondents (5.5% response rate), who are more likely to be older, female, living in less socioeconomically deprived areas, and reporting fewer physical and mental health conditions than the general population in the UK [101, 103]. Many studies have shown that this “healthy volunteer bias” distorts the associations among phenotypes [104–106], and with genetic variants [107] that are associated with self-selection. Notably, several genetic variants that are associated with self-selection are also associated with psychiatric disorders [108–111]. Unless adequately mitigated through statistical approaches [104, 112–115] or validated through experimental means [112], genetic findings from volunteer samples may compound biases [104]. Despite these limitations, population-based biobanks have made important contributions to progress in psychiatric genetics.

### Control ascertainment

While the recruitment and assessment of cases dominate ascertainment debates, the selection of controls poses underappreciated methodological issues [116–118]. In clinical ascertainment, case and control participants are typically recruited independently, so case-control differences may be driven by both disease liability and ascertainment procedures. While the ascertainment biases discussed above regarding the selection of cases apply to the selection of controls in a broad sense, there are several control specific ascertainment factors that deserve attention. Most importantly, to identify meaningful case-control differences, controls should resemble cases in all characteristics *except* for the absence of the disorder for which cases are selected. Controls selected on this principle are referred to as *normal* controls.

However, the collection of controls in many genetic studies does not follow this principle, and the strategies used are not always adequately reported [74, 75]. In particular, many psychiatric GWAS use *super-normal* controls who are screened for the disorder being studied *and* other psychiatric disorders that are not screened out of cases [119, 120]. Epidemiological studies have shown that the use of super-normal controls not only exaggerates case-control differences but can induce familial/genetic correlations in the absence of any true relationships [120]. In family studies, the use of super-normal controls produces spurious co-aggregation between disorders, with the magnitude of the bias increasing proportional to the population prevalence of screened-out correlated disorders [121]. Simulation studies demonstrate that the symmetrical use of super-normal controls in GWAS of two disorders inflates rG proportional to the population prevalence of the two disorders and the simulated magnitude of the association [122]. For example, if parallel GWASs of MDD and SUD were conducted that included the opposite disorder in the cases but excluded them from the controls, the resulting MDD-SUD rG estimate would be overestimated.

The problem here, simply put, is the case-vs-super-normal-control difference reflects *not only case-control differences for the target disorder but also of any traits or diseases that were asymmetrically screened out of the control group*. This will upwardly bias GWAS effect sizes as a function of the prevalence of the diseases that are disproportionately screened out of the controls, compounding biases in analyses that use the summary statistics [122]. To further complicate the situation, some studies not only screen controls based on their own phenotype but also on the phenotypes of close relatives [123]. Alternatively, because screening potential controls can be effortful and expensive, unscreened controls have been used in some psychiatric GWAS [124, 125]. In this scenario, the control group may contain cases of the target disorder at approximately the population prevalence. Here, without appropriate correction, genetic associations are downwardly biased, with the magnitude of the bias increasing for more prevalent disorders in the population [126].

## Going forward

In GWAS meta-analyses, most of the samples for common disorders (e.g., MDD) are population-ascertained with shallow phenotyping, whereas those for less common disorders (e.g. SZ, BD) are predominantly clinically ascertained or obtained through EHRs and registries. Thus, biases in GWAS meta-analysis may operate differently across disorders. This complicates cross-disorder analyses, where shared genetic effects across disorders may reflect an unknown mixture of biases due to the different assessment and ascertainment strategies and true etiologic overlap between diagnostic entities. While misdiagnosis influences rGs between genetically related disorders [127], simulation studies suggest that an implausibly high level of misdiagnosis [3] would be required to account for the observed rGs between most pairs of psychiatric disorders in the absence of true genetic overlap. Nevertheless, lower levels of case misclassifications can inflate rG especially when misdiagnosis occurs for both disorders, and the magnitude of inflation depends on the magnitude of the rGs between disorders [45] and their prevalence. Finally, inflation of rGs can result from other sources including cross-trait assortative mating [128]. While some of these biases may cancel each other out, accurately identifying the source of pleiotropy and comorbidity remains essential for illuminating the shared genetic architecture of psychiatric disorders. In this section, we summarize ways to reduce or quantify biases that affect assessment and ascertainment strategies in both individual and cross-disorder genetic findings and give recommendations for future data collection efforts.

### Refining phenotypes

Phenotypic quality control substantially increases the validity of psychiatric diagnoses, including applying stringent clinical criteria [45], requiring multiple endorsements from different assessment strategies [49, 129], and ensuring consistency of endorsements across time [130]. For example, correcting for mis-reports in different measures of alcohol use increases the rGs across different assessment strategies from 0.79 to >0.9 [130].

We now have a wide range of tools to quantify and compare the genetic architectures of the same disorder collected through different assessment and ascertainment strategies [131]. At the individual locus level, we can assess the replicability or heterogeneity of effects across assessment strategies [19, 28, 132]. At the genome-wide level, we can assess whether SNP-heritability estimates of the same disorder are similar across different study designs, and whether rGs among them are close to unity [10, 45, 62]. We can further assess whether polygenic risk scores (PRSs) from each assessment or ascertainment strategy robustly associate with scores from the other strategies [8, 62]. A recently derived metric called PRS Pleiotropy takes these approaches further, by assessing how well a PRS predicts the disorder of interest relative to other phenotypes (available in biobanks and EHRs) [133]. With PRS Pleiotropy as a means to assess specificity, we can identify clinically valid shallow phenotypes (e.g. clozapine treatment for SZ [18, 27, 28]) to include in GWAS meta-analyses. While no single test provides unambiguous evidence of bias, consistency across multiple tests provide convergent evidence of stable genetic effects.

We can also utilize statistical methods that combine genetic effects from shallow and deep measures to maximally leverage all data collected for improving GWAS power while maintaining reasonable specificity. These methods include LT-FH [134] (which models family history-based liability to disease), MTAG [135] (a meta-analytical approach leveraging information from collateral GWAS phenotypes with high rG to target GWAS), and Genomic SEM [136] (a framework for modeling genetic covariance structure that can be used to specify common and unique genetic factors underlying a system of GWAS phenotypes and perform GWAS discovery on those factors). In contrast to methods that require carefully choosing input phenotypes, multiple-phenotype imputation presents a relatively agnostic way to boost sample sizes for deep measures of a disorder (usually available in only a subset of individuals in a biobank) [133, 137]. Exploring different imputation approaches, especially non-linear models, can further allow us to utilize more data modalities (multi-omics [138–141], imaging [142, 143], data from smartphones and wearable devices [144, 145]). Further methodological developments applied to time-censored and longitudinal data in EHRs may help to refine diagnostic accuracy beyond missing value imputation [29, 92].

### Accounting for ascertainment biases

As biases are prevalent and unavoidable, developing methods to assess and control for them is critical for obtaining generalizable findings [96]. One way to address known bias, such as sex-differential participation, is to stratify GWAS and all subsequent analyses by the known factor [114, 146] However, psychiatric disorders and relevant comorbid traits are unlikely to be biased by a single factor as straightforward as sex-differential participation, and stratification by factors that are also genetically regulated may induce collider biases [107, 113].

Several studies have proposed the use of inverse probability (IP) weightings (up-weighting participants with features identified to be associated with lower participation) [113, 147, 148] to improve representativeness of relationships identified between variables of interest (and interactions between them) in participants of volunteer-based biobanks [96, 104, 146]. This approach has been shown to improve the robustness of GWAS findings, rGs, and results of Mendelian randomization (MR) [115]. Notably, IP weighting relies on training feature selection models using variables affecting participation that are available in both the unrepresentative dataset (e.g., the UK Biobank) and a representative dataset from the same population (e.g. the UK Census microdata [104]). As misspecification of IP weightings may introduce further biases [113], feature selection for IP models will vary across different psychiatric disorders based on disease severity and other known risk factors [115, 128, 130, 146]. Further, under some circumstances IP weighting may reduce power [149]. Despite these limitations, this approach can be applied to correct for participation biases in EHRs and cohort studies. Of note, as we move towards analyzing disease trajectories that involve diagnostic conversions and comorbidities, we need to address a specific form of ascertainment bias: the index event bias [113]. For example, genetic effects identified as associated with late-onset BD (the disease incidence) in MDD cases would be biased by genetic effects associated with MDD diagnosis (the index event) [150, 151]. However, their utility in investigations into comorbidities among psychiatric disorders are limited, as they assume no correlation or interaction between SNP effects on disease progression and incidence. Methods for identifying, clustering, and correcting for incidence have been developed [152, 153], but like IP weighting methods, they are currently low in power.

Quantifying and correcting for ascertainment biases is an active area of research [113]. Nevertheless, novel methods are likely to remain imperfect. As such, sensitivity analyses of genetic associations are recommended to identify the bounds of worst-case biases and the minimal level of bias necessary to account for the genetic findings [154].

### Investigating disease trajectories and comorbidities from a genomic perspective

While most psychiatric disorders have clear developmental components, developmental processes are just beginning to be integrated into genomic analyses. Genetic studies of disease trajectories have become more feasible with the increased availability of data from biobanks, EHRs and registries linked with genetic data that may inform the interrelated development of multiple disorders. Self-reports of first diagnosis from the UK Biobank [155], for instance, enable the examination of temporal factors that may affect the comorbidity between symptom criteria for anxiety disorders and MDD [156] as well as their comorbidities with non-psychiatric phenotypes [157, 158]. Alternatively, repeated measurements from EHR or registry records provide the longitudinal elements necessary for prospective genomic studies [159, 160]. Furthermore, there are now large genotyped prospective samples, not relying on retrospective data [161, 162].

When considering the trajectory of disease progression, how patients are sampled also has major implications for genetic analyses and comorbidity. A recent longitudinal Swedish study of cases of MDD, BD, and SZ (using recorded discharges from the Swedish registry) concluded that “Over time clinical diagnosis and genetic risk profiles became increasingly consilient [58]”. These results suggest that genetic correlations between BD and SZ may be higher in cases examined early versus later in their course of illness. What might be termed diagnostic error could in part reflect the clinical development of the disorders over time [59, 60].

Records of clinical diagnoses of psychiatric disorders from millions of individuals in the Swedish and Danish registries have shown high, though variable, rates of comorbidities between different pairs of psychiatric disorders [163–165], corroborated by findings from a Columbian EHR study [147]. Studies using polygenic risk scores (PRS) [166, 167] or family genetic risk scores (FGRS) [168–171] can investigate patterns of shared genetic risk between pairs of disorders or their comorbidities. Many interesting insights confirm previous expectations: FGRS of disorders vary in their ability to predict comorbid disorders as would be expected from variation in the prevalence of individual disorders and genetic correlations between them [165]; MDD cases with higher FGRS for BD have an elevated rate of conversion to a BD diagnosis (also generally true for other pairs of disorders) [58]; multinomial logistic regression using both PRS and FGRS are able to identify genetic heterogeneities among cases of MDD [170] and ADHD with different comorbid disorders [166]. Some findings, however, defy previous expectations and offer new opportunities for expanding our understanding of psychiatric disorders: other non-affective psychoses are found to have much lower SZ FGRS than expected, calling into question their inclusion in SZ analyses [168]. To date, psychiatric GWAS has not typically stratified analyses by different patterns of comorbidity. Following from the PRS and FGRS genetic heterogeneity results, this reflects a promising avenue for future cross-disorder genomic research to evaluate the extent to which different comorbid presentations implicate unique biological pathways.

Most psychiatric genetic studies to date have taken a cross-sectional disease-centric approach, focusing on investigations into genetic contributions to individual disorders while ignoring current comorbidities or subsequent conversions to other disorders. We would hypothesize that phenotypes that share similar trajectories also share genetic (in addition to environmental) precursors. Not all diagnostic switches (defined to be conversions among disorders that are exclusion criteria for each other in the DSM [172]) may pass this validity test, as they are based entirely on DSM-defined exclusion criteria that may be arbitrary. Disease trajectory analyses, therefore, present important opportunities for improving and refining disease nosology and DSM criteria. In fact, taking the trajectory-centric approach may enable us to get traction on potential biases that might otherwise inflate (or deflate) estimates of apparent pleiotropy, such as cross-sectional misclassifications of two diagnoses with frequent transitions [173] (e.g. BD and MDD, psychotic disorders and affective psychoses), and age-related differences in genetic correlations. Accordingly, we need strategies for keeping analyses tractable without losing resolution. This may require identifying biologically interesting questions, defining relevant phenotypes [58], designing useful data formats [164], and developing necessary statistical metrics [174]. Statistical approaches developed for assessing multimorbidity across the entire disease classification tree, currently employed on first-diagnoses or inpatient data in the UK Biobank, may also be customized to accommodate diagnostic criteria specific to psychiatric disorders, or longitudinal trajectory data in EHRs and registries [175–177].

### Recommendations for future data collections

Integrating data from disparate assessment and ascertainment strategies will continue to pose challenges to psychiatric genetics in the foreseeable future. While little can be done to alter the study design choices of existing data, we hope that in planning future genomic data collection efforts, researchers will consider the implications that assessment and ascertainment techniques have on the validity, severity, comorbidity and genetic sharing across psychiatric disorders.

Diagnostic validity for individual disorders is a necessary but insufficient condition for any phenotyping approach. Cases and controls in new cohorts, especially when collected through different strategies, should demonstrate similar epidemiological relationships with known risk and protective factors in the population they are obtained from. For example, SZ cases should show a range of characteristics including male excess, mean age of onset in early to mid-20s, and present evidence of poor premorbid social or educational functioning and impaired social functioning, in addition to the canonically assessed key symptoms. Further, tests of the specificity of identified genetic risk (see above) are also critical.

Deep phenotyping studies will play a vital role in dissecting and understanding findings from heterogeneous meta-analyses, buttressing the translation of psychiatric molecular genetic results into diagnostic and treatment regimens. This is particularly important for cross-disorder genetic studies, as shallow phenotyping may be less accurate for some disorders than others. For these, we recommend: (i) expanding symptom assessment beyond DSM or ICD criteria to permit the measurement of other relevant clinical and non-clinical dimensions and/or subtypes that may not be captured by standard criteria, (ii) hiring trained mental health interviewers familiar with the relevant symptomatology of the case sample, (iii) establishing rigorous quality control procedures for interviewers such as monitored interview recordings by trained editors, and (iv) where possible, especially for more severe disorders, complementing interviews with reviews of relevant clinical records. For such studies we would also recommend consensus all-sources diagnostic procedures.

Conversely, studies that use non-clinician assessment approaches will continue to play a key role in recruiting large samples that are necessary for genomic analyses. For these studies, we recommend: (i) avoiding single item screens and prior treatment- or diagnosis-based questions (e.g., “Have you ever been diagnosed with …”) in favor of brief self-report versions of full diagnostic criteria, some of which have been validated in genetic designs [178, 179]; (ii) remaining cognizant of the potential for misdiagnosis especially with regard to false positives and negatives for standard screens for psychotic symptoms [180]; (iii) recognizing the impact of the time-frame of assessment, recalling that, overall, lifetime measures are likely to be more genetically informative, and (iv) utilizing modular assessment designs that allow participants to be recontacted to obtain more detailed assessments where necessary or followed-up for longitudinal assessments and trajectory analyses.

Selecting ascertainment strategies for psychiatric genomic investigations will likely be guided by the researcher’s access to data. However, it is important to keep the corresponding ascertainment biases in mind when analyzing genomic data. Furthermore, we recommend (i) using representative (not super-normal) controls, (ii) developing an ascertainment frame for cases that avoids oversampling severe and/or treatment resistant illness unless that is a specific focus of the design, and (iii) when possible, assessing phenotypes through measurement non-invariance techniques.

Finally, we call for greater efforts recruiting cohorts from diverse ancestries and environments. Most genetic studies have been performed on individuals of European descent who have relatively easy access to healthcare. Not only do we need to increase data collection in previously underrepresented communities, we must also pay careful attention to the translation of assessment instruments and, where necessary, design and benchmark new data collection protocols to address language and cultural differences. Further, with the increasing use of electronic health records in genetic research, we would like to urge the greater research community, not just in psychiatric genetics [82, 181–183], to investigate the social determinants that bias representation of different communities in these resources [184, 185]. Such biases can skew our understanding of disorder risk and comorbidities, and if uncorrected, result in increasing healthcare disparities [186].

## Conclusions

Over the last 15 years, robust genetic associations have been identified for numerous psychiatric disorders, both under the auspices of the PGC and in independent studies. As we move into an era of historically large sample sizes in the genomic sciences, it is essential that we avoid assuming that larger samples will overcome biases and remain vigilant to the challenges associated with various measurement and ascertainment approaches in studies contributing to large meta-analyses. The translation of genetic findings into novel diagnostic techniques and treatment regimens for psychiatric disorders are predicated on valid assessment techniques and unbiased ascertainment strategies, as well as statistical methods to analyze genomic data. The aim, which we should always keep in mind, is identifying loci affecting risk for the disorders and disaggregating pleiotropic from disorder-specific variants. This will enable us to understand the biological mechanisms of individual psychiatric disorders and their comorbidity and serve as the foundation for improvements in diagnoses and individualized treatments of patients living with mental illness.
