# Correction: Assessment and ascertainment in psychiatric molecular genetics: challenges and opportunities for cross-disorder research

**DOI:** 10.1038/s41380-025-02914-4

**Published:** 2025-01-28

**Authors:** Na Cai, Brad Verhulst, Ole A. Andreassen, Jan Buitelaar, Howard J. Edenberg, John M. Hettema, Michael Gandal, Andrew Grotzinger, Katherine Jonas, Phil Lee, Travis T. Mallard, Manuel Mattheisen, Michael C. Neale, John I. Nurnberger, Wouter J. Peyrot, Elliot M. Tucker-Drob, Jordan W. Smoller, Kenneth S. Kendler

**Affiliations:** 1 Helmholtz Pioneer Campus, Helmholtz Munich, Neuherberg, Germany; 2 Computational Health Centre, Helmholtz Munich, Neuherberg, Germany; 3 School of Medicine and Health, Technical University of Munich, Munich, Germany; 4 Department of Psychiatry and Behavioral Sciences, Texas A&M University, College Station, TX USA; 5 Centre of Precision Psychiatry, University of Oslo, Oslo, Norway; 6 Division of Mental Health and Addiction, Oslo University Hospital, Oslo, Norway; 7 KG Jebsen Centre for Neurodevelopmental disorders, University of Oslo, Oslo, Norway; 8 Department of Cognitive Neuroscience, Donders Institute for Brain, Cognition and Behavior, Radboud University Medical Center, Nijmegen, The Netherlands; 9 Karakter Child and Adolescent University Center, Nijmegen, The Netherlands; 10 Department of Biochemistry and Molecular Biology, Indiana University School of Medicine, Indianapolis, IN USA; 11 Department of Medical and Molecular Genetics, Indiana University School of Medicine, Indianapolis, IN USA; 12 Virginia Institute for Psychiatric and Behavioral Genetics, Virginia Commonwealth University, Richmond, VA USA; 13 Departments of Psychiatry and Genetics, University of Pennsylvania, Philadelphia, PA USA; 14 Lifespan Brain Institute at Penn Med and the Children’s Hospital of Philadelphia, Philadelphia, PA USA; 15 Institute for Behavioral Genetics, University of Colorado Boulder, Boulder, CO USA; 16 Department of Psychology and Neuroscience, University of Colorado Boulder, Boulder, CO USA; 17 Department of Psychiatry & Behavioral Health, Stony Brook University, Stony Brook, NY USA; 18 Center for Genomic Medicine, Massachusetts General Hospital, Boston, MA USA; 19 Department of Psychiatry, Harvard Medical School, Boston, MA USA; 20 Center for Precision Psychiatry, Department of Psychiatry, Massachusetts General Hospital, Boston, MA USA; 21 Psychiatric and Neurodevelopmental Genetics Unit, Center for Genomic Medicine, Massachusetts General Hospital, Boston, MA USA; 22 Department of Community Health and Epidemiology and Faculty of Computer Science, Dalhousie University, Halifax, NS Canada; 23 Institute of Psychiatric Phenomics and Genomics (IPPG), University Hospital of Munich, Munich, Germany; 24 Department of Biomedicine, Aarhus University, Aarhus, Denmark; 25 Department of Psychiatry, Virginia Commonwealth University, Richmond, VA USA; 26 Department of Psychiatry, Indiana University School of Medicine, Indianapolis, IN USA; 27 Stark Neurosciences Research Institute, Indiana University School of Medicine, Indianapolis, IN USA; 28 Department of Psychiatry, Amsterdam UMC, Vrije Universiteit, Amsterdam, The Netherlands; 29 Amsterdam Public Health, Amsterdam UMC, Vrije Universiteit, Amsterdam, The Netherlands; 30 Department of Psychology, University of Texas at Austin, Austin, TX USA; 31 Stanley Center for Psychiatric Research, Broad Institute of MIT and Harvard, Cambridge, MA USA

**Keywords:** Genetics, Diagnostic markers

Correction to: *Molecular Psychiatry* 10.1038/s41380-024-02878-x, published online 27 December 2024

In this article the author’s name Wouter J. Peyrot was incorrectly written as Wouter Peyrout.

The original article has been corrected.

